# Improved Bioavailability of Poorly Water-Soluble Drug by Targeting Increased Absorption through Solubility Enhancement and Precipitation Inhibition

**DOI:** 10.3390/ph14121255

**Published:** 2021-12-02

**Authors:** Ju-Hyun Lee, Chulhun Park, Kwon-Yeon Weon, Chin-Yang Kang, Beom-Jin Lee, Jun-Bom Park

**Affiliations:** 1 College of Pharmacy, Sahmyook University, Seoul 01795, Korea; jelly_3004@naver.com (J.-H.L.); kangjy@syu.ac.kr (C.-Y.K.); 2 Faculty of Pharmacy & Pharmaceutical Sciences, University of Alberta, Edmonton, AB T6G 2E1, Canada; chulhun@ualberta.ca; 3 College of Pharmacy, Catholic University of Daegu, Gyeongsan-si 38430, Korea; weonky@cu.ac.kr; 4 College of Pharmacy, Ajou University, Suwon 16499, Korea; bjl@ajou.ac.kr; 5 Bioavailability Control Lab, Sahmyook University, Seoul 01795, Korea

**Keywords:** hot-melt extrusion, itraconazole, solid dispersion, modified drug release, absorption area

## Abstract

Itraconazole (ITZ) is a class II drug according to the biopharmaceutical classification system. Its solubility is pH 3-dependent, and it is poorly water-soluble. Its pKa is 3.7, which makes it a weak base drug. The aim of this study was to prepare solid dispersion (SD) pellets to enhance the release of ITZ into the gastrointestinal environment using hot-melt extrusion (HME) technology and a pelletizer. The pellets were then filled into capsules and evaluated in vitro and in vivo. The ITZ changed from a crystalline state to an amorphous state during the HME process, as determined using DSC and PXRD. In addition, its release into the gastrointestinal tract was enhanced, as was the level of ITZ recrystallization, which was lower than the marketed drug (Sporanox^®^), as assessed using an in vitro method. In the in vivo study that was carried out in rats, the AUC_0–48h_ of the commercial formulation, Sporanox^®^, was 1073.9 ± 314.7 ng·h·mL^−1^, and the bioavailability of the SD pellet (2969.7 ± 720.6 ng·h·mL^−1^) was three-fold higher than that of Sporanox^®^ (*** *p* < 0.001). The results of the in vivo test in beagle dogs revealed that the AUC_0–24h_ of the SD-1 pellet (which was designed to enhance drug release into gastric fluids) was 3.37 ± 3.28 μg·h·mL^−1^ and that of the SD-2 pellet (which was designed to enhance drug release in intestinal fluids) was 7.50 ± 4.50 μg·h·mL^−1^. The AUC of the SD-2 pellet was 2.2 times higher than that of the SD-1 pellet. Based on pharmacokinetic data, ITZ would exist in a supersaturated state in the area of drug absorption. These results indicated that the absorption area is critical for improving the bioavailability of ITZ. Consequently, the bioavailability of ITZ could be improved by inhibiting precipitation in the absorption area.

## 1. Introduction

Biopharmaceutical classification system (BCS) class II drugs have low solubility and high permeability; however, their bioavailability can be sufficiently increased through solubilization. Amorphous solid dispersion (SD) is a widely feasible formulation technique for improving the apparent solubility of poorly water-soluble drugs [1]. Amorphous SDs have higher free energy than crystalline active pharmaceutical ingredients. They exhibit high solubility and supersaturation in the gastrointestinal environment. However, they are thermodynamically unstable and generally precipitate over time. As a result, the solubility of amorphous SDs decreases without reaching equilibrium. In addition, supersaturation and precipitation are also influenced by several physiological factors, including pH, gastric emptying rate, and the composition of the simulated biological fluid [2,3,4]. Therefore, the bioavailability of poorly water-soluble drugs can be increased by preparing an SD using a polymer that suppresses precipitation while maintaining a higher supersaturation state in the gastrointestinal tract [5,6].

Hydrophilic polymers, such as Soluplus^®^, hydroxypropyl methylcellulose phthalate (HPMCP), and polyvinyl alcohol (PVA) enhance supersaturation and inhibit precipitation by employing hydrophobic interactions with drugs or intermolecular interactions, such as hydrogen bonds, ionic bonds, and van der Waals interactions [7,8,9,10]. Solvent evaporation and melting are mainly used to prepare amorphous SDs. Solvent evaporation involves dissolving a poorly water-soluble drug and a polymer (or polymers) using an organic solvent and then evaporating the solvent to prepare an SD at a relatively low temperature [11]. The melting method involves preparing a dispersion of a poorly water-soluble drug and a polymer (or polymers) in a molten state at a high temperature; hot-melt extrusion (HME) is one such representative technology.

HME is a promising SD technology in the pharmaceutical industry owing to its ease of application, economic benefits, and speed. Moreover, it does not require organic solvents [12,13,14,15,16]. Currently, Kaletra^®^, Eucreas^®^, Noxafil^®^, and Viekira™ are commercially available drugs that are manufactured using the HME technology that has been applied for SD preparation and targeted drug delivery, sustained release, and taste masking [17,18]. HME technology is also applied downstream when manufacturing various types of pharmaceutical formulations, such as pellets, films, granules, tablets, implants, stents, transdermal absorbents, and 3D printing filaments [19,20,21].

Itraconazole (ITZ) is a broad-spectrum antifungal agent used to treat *Candida vaginitis*, corpus callosum, ringworm infection, oral candidiasis, and fungal keratitis. It is a weak base with a pKa of 3.7 and displays an increase in solubility in gastric juice because of its low pH. However, the ITZ that is dissolved in gastric juice presents reduced solubility as it moves toward the intestine, causing its precipitation in intestinal fluids [22,23,24]. Several studies have investigated the effect of excipients on hot-melt extrusion formulations of ITZ [25,26]. Most of them have mainly focused on improving the dissolution rate of ITZ by selecting a suitable polymer [27]. The limited pH solubility of ITZ means that it can only achieve a narrow absorption area in the gastrointestinal tract. Considering the narrow absorption window of ITZ, we hypothesized that the combination of different HME pellets with their various ITZ release behaviors could help us fine-tune the bioavailability of ITZ, leading to the better administration of ITZ compared to a single HME pellet. A clear mechanistic study that assesses ITZ absorption behaviors in the supersaturated phase during continuous dissolution has not been conducted. Therefore, ITZ was considered to be a model drug for this study.

In this study, ITZ, a poorly water-soluble BCS II drug, was used as the model drug. This study aimed to develop ITZ SD pellets using HME technology in order to maximize the bioavailability of ITZ in biological systems. The physicochemical properties of the ITZ SD pellets were evaluated using FE-SEM, DSC, PXRD, and FT-IR. The ITZ release rates of the physical mixture (PM) and SD were determined using the pH-shifting method of the dissolution study. Furthermore, the dissolution behavior of ITZ was investigated based on its appearance and XRD patterns. The stability of ITZ SD formulations was monitored for 6 months under long-term and accelerated conditions using a well-closed container. Pharmacokinetic (PK) studies of ITZ formulations were performed on rats and beagle dogs.

## 2. Results and Discussion

### 2.1. Assessment of Drug Contents, Release Behaviors, and Morphological Changes

**Figure 1:**
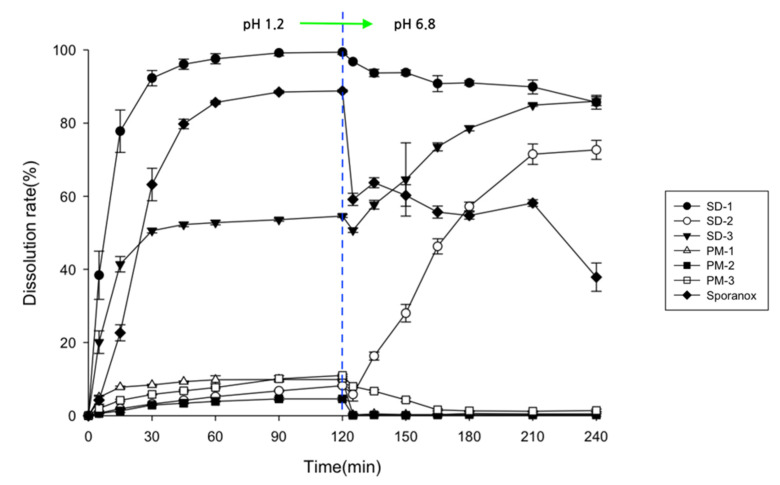
Drug release behaviors of SDs, PMs, and Sporanox^®^ in pH-shift dissolution medium (*n* = 6).

The SD pellets were prepared using PVA (Parteck^®^ MXP, which has excellent HME processability) [28], HPMCP (which can inhibit drug recrystallization) [29], and Soluplus^®^ (which has excellent solubilization abilities) [30]. Two types of ITZ SDs with hydrophilic carriers were prepared. SD-1 constituted the PVA-based polymer, prepared with Parteck^®^ MXP to enhance the solubility of ITZ in the stomach. SD-2 was prepared using a ternary mixture of HPMCP HP-55, Soluplus^®^, and ITZ. It was designed to increase the solubility and inhibit the precipitation of ITZ in the small intestine. The contents of the drugs in SD-1 and SD-2 that were prepared using the HME technology were 99.2 ± 2.5% and 98.4 ± 3.1%, respectively, and the SDs did not decompose or degrade. In addition, drug release from the SDs, their PMs, and commercial ITZ (Sporanox^®^) are shown in Figure 1. In the dissolution test, there were significant differences in the dissolution rate of ITZ between the PM and SDs. Therefore, it was confirmed that the solubility enhancement of ITZ had been achieved using HME technology with different carriers.

The dissolution rate of the crystalline ITZ powder was less than 1.5%, regardless of the pH of the dissolution medium (data not shown). Sporanox^®^ is an ITZ reference product that is prepared using a spray–drying process with hypromellose. The dissolution rate of Sporanox^®^ reached a maximum of 80% after 2 h in gastric fluid (pH 1.2) but decreased to less than 60% in intestinal fluid (pH 6.8). These results indicated that the ITZ in Sporanox^®^ was highly dissolved at a low pH in the gastric environment and rapidly recrystallized when the pH increased to over 6.8 [31]. Capsules with the SD-1 pellets showed a dissolution rate of 77.8 ± 5.8% after 15 min in simulated gastric fluid. As a result, it had released up to 96.1 ± 0.6% of the drug after 45 min. After changing the pH from 1.2 to 6.8, the dissolution rate of ITZ in the SD-1 capsule gradually decreased due to ITZ precipitation. The ITZ precipitation of SD1 was slower than that of Sporanox^®^.

The recrystallization of the ITZ in the SDs that had been prepared with other hydrophilic polymers has been reported to occur even in the gastric environment [32,33]. However, the pellets prepared using the HME method with PVA did not precipitate or recrystallize in the gastric environment in this study. These results demonstrated that the PVA in the SDs that were prepared using HME technology inhibits ITZ recrystallization in gastrointestinal environments. Drug release from capsules with SD-2 pellets was less than 9% after 2 h in the gastric medium and increased in the small intestinal medium (pH 6.8), reaching 46.3 ± 2.1% after 45 min and 72.7 ± 2.6% after 120 min. While SD-1 showed a rapid drug release pattern in the gastric medium, SD-2 was designed to be released in the small intestine media.

The SD-3 capsules showed a continuous drug release pattern regardless of the pH. The ITZ in SD-1 was dissolved in the simulated gastric fluid. The SD-2 pellets gradually dissolved as the pH increased. In addition, no precipitation was observed in the SD-3 capsules. As the pH of the medium increased, the precipitation of ITZ in SD-1 was masked relatively well by the disintegration of SD-2. We found that the ITZ in the SD-3 capsules was continuously released into the gastrointestinal tract, thereby maintaining a supersaturated state.

**Figure 2:**
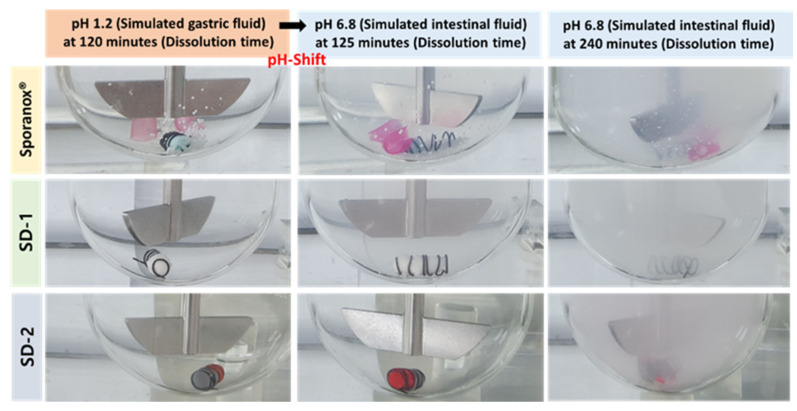
Visual observation of the release behaviors of the ITZ formulations (Sporanox^®^, SD-1, and SD-2) in the pH-shifting method.

**Figure 3:**
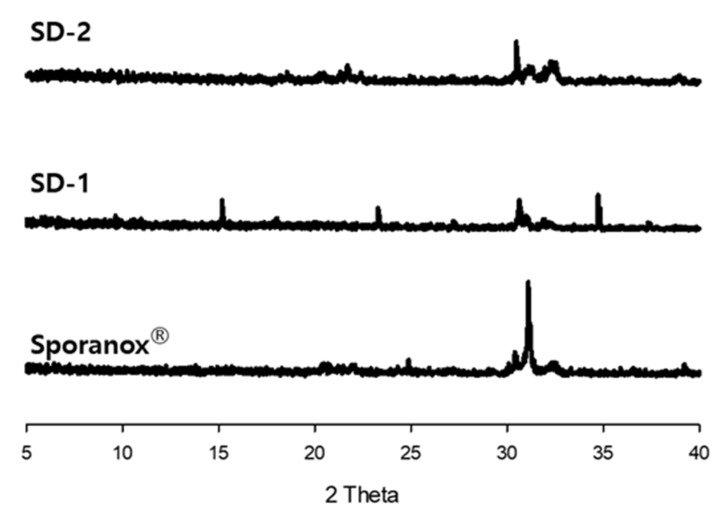
X-ray diffraction patterns of dried ITZ samples after the dissolution study.

Furthermore, the clarity of the dissolution medium changed after the pH of the dissolution was changed. A colorless and transparent medium was observed at a pH of 1.2. After the medium changed from pH 1.2 to pH 6.8, the medium gradually became opaque and white (Figure 2). The dissolution samples were then dried at 60 °C. As shown in Figure 3, each sample exhibited different XRD patterns. The differences in ITZ solubility induced changes in the turbidity of the dissolution medium. A strong X-ray diffraction peak was observed at around 32° for ITZ, while weak diffraction peaks at 15° and 23° were observed for Sporanox^®^. Peaks were observed at 32° and 35° for SD-1 pellets. These results suggest that the dissolution rate of Sporanox^®^ and SD-1 pellets decreased at pH 6.8, owing to the recrystallization of ITZ. In addition, relatively weak diffraction peaks were observed at approximately 30° and 32° for the SD-2 pellets, and it was confirmed that in the SD-2 pellets, the level of ITZ recrystallization in the intestinal environment was lower than that for the SD-1 pellets and Sporanox^®^.

### 2.2. Physicochemical Properties of ITZ SD

**Figure 4:**
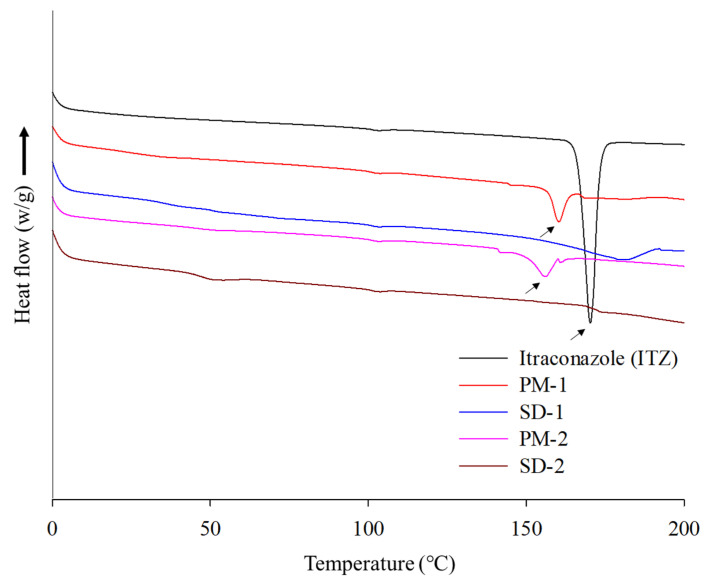
DSC thermograms of itraconazole (ITZ), PM-1, SD-1, PM-2, and SD-2.

**Figure 5:**
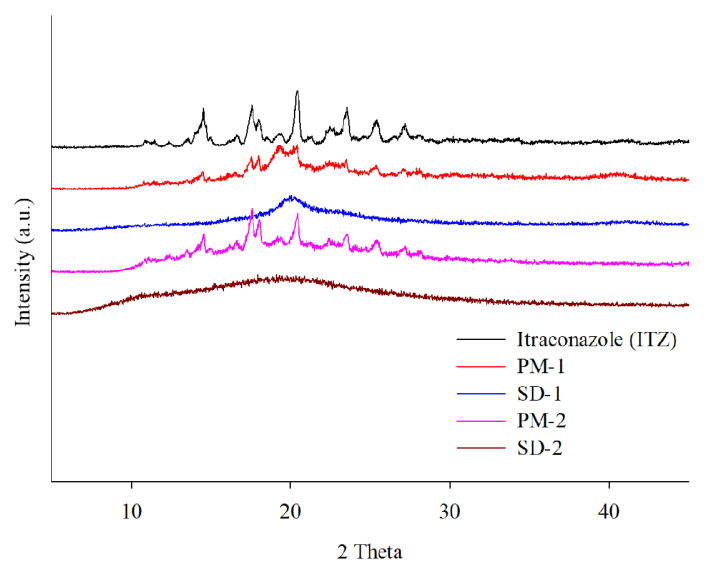
X-ray diffraction patterns of itraconazole (ITZ), PM-1, SD-1, PM-2, and SD-2.

DSC and PXRD analyses were performed to confirm the change of the hot-melt extrudates from the crystalline to the amorphous form. DSC analysis revealed an endothermic peak at 167 °C, which is the melting point of ITZ, but such an endothermic peak was not observed in SD-1 and SD-2 (Figure 4). These results indicated that the crystalline ITZ changed to an amorphous SD upon undergoing the HME process, indicating that the drug was evenly dispersed in the polymer [34]. The PXRD analysis showed sharp peaks between 10° and 30° for ITZ, indicating that the drug was present in a crystalline form. Crystalline peaks were not observed for the SD-1 and SD-2 pellets, and it was confirmed through the XRD and DSC analyses that the crystal structure of ITZ changed to an amorphous form in the SD that had been prepared using HME technology (Figure 5) [35].

**Figure 6:**
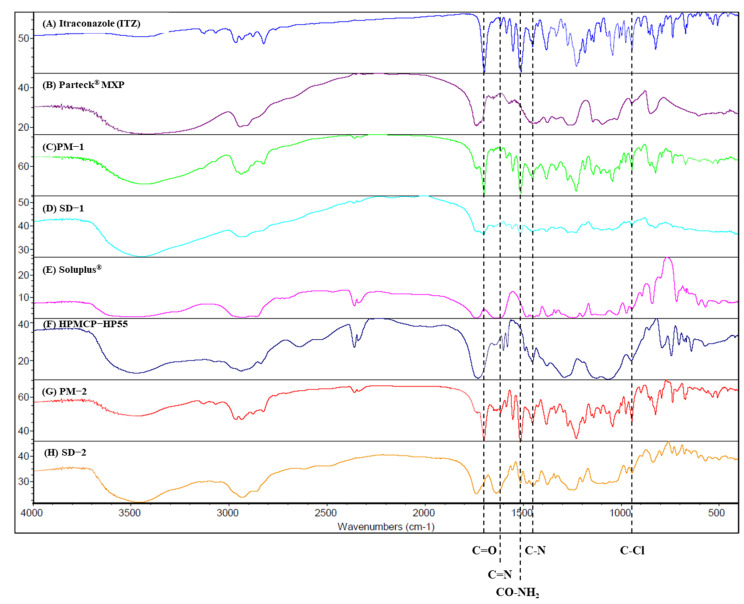
FT-IR spectra of: (**A**) itraconazole, (**B**) Parteck MXP, (**C**) PM-1, (**D**) SD-1, (**E**) Soluplus^®^, (**F**) HPMCP-HP55, (**G**) PM-2, and (**H**) SD-2.

FT-IR was performed to confirm the interaction between ITZ and the polymers (Figure 6). Distinct absorption bands of ITZ were observed at 944.9 cm^−1^ (970~920 cm^−1^, trans = C–H out-of-plane bending), 1450.9 cm^−1^ (C-N triazole), 1510.2 cm^−1^ (CO-NH_2_), 1613.8 cm^−1^ (1650~1610 cm^−1^, C=C stretching), and 1698.1 cm^−1^ (1710~1680 cm^−1^, C=O stretching) [36]. In the PVA (Parteck^®^ MXP), a peak was observed at 3418.4 cm^−1^ that was related to OH stretching. In contrast, another peak for the PVA was observed at 1738.4 cm^−1^, which was related to the unhydrolyzed ester group. The peak of SD-1 was observed to shift to 3445.7 cm^−1^, which seems to represent a hydrogen bond between ITZ and PVA. This hydrogen bond improves the stability of the SD [37]. Soluplus^®^ showed distinct absorption bands at 3462.5 cm^−1^ (O-H) and 1443.8 cm^−1^ (C-O-C) [38]. HPMCP-HP55 showed a distinct band at 1732.3 cm^−1^, which may be due to the symmetric and asymmetric stretching vibrations of the C=O group [39]. The ITZ has acceptor hydrogen bond groups in its structure.

Additionally, Soluplus^®^ has an ether, hydroxyl, and carbonyl group that can donate a proton to the hydroxy groups, while HPMCP has a phthalate functional group that can act as the proton-accepting polymer. As a result, hydrogen bonds can be formed in SD-2 because both the proton donor and acceptor groups coexist. The peak shift of SD-2 was shown to be at 3447.9 cm^−1^ [40]. This hydrogen bonding triggered drug release while inhibiting the recrystallization of ITZ, even when the pH of the dissolution medium was increased.

**Figure 7:**
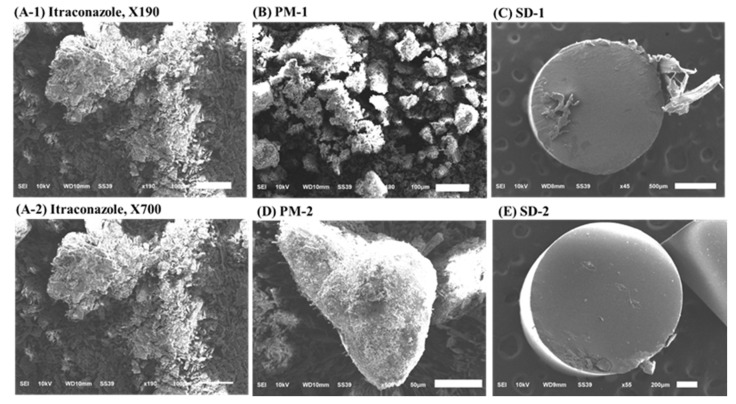
Scanning electron microscopy (SEM) images of: (**A-1**) itraconazole (ITZ) (190× magnification), (**A-2**) itraconazole (ITZ) (700× magnification), (**B**) PM-1, (**C**) SD-1, (**D**) PM-2, and (**E**) SD-2.

SEM measurements revealed crystal forms on the surface of the ITZ powder and PMs. However, in the cross-section of the SD-1 and SD-2 pellets, the crystal form of the drug was not observed. It was confirmed that the drug and polymer were evenly distributed (Figure 7).

### 2.3. Stability Test

**Figure 8:**
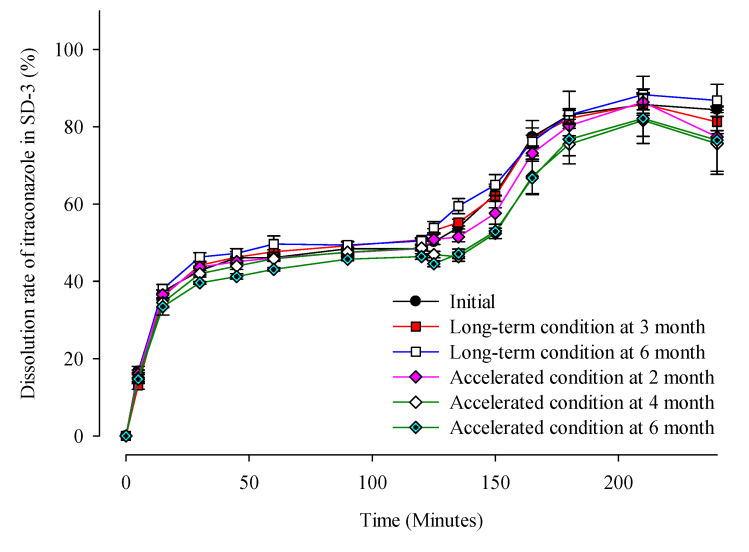
Dissolution profiles of ITZ in SD-3 capsules during the stability test.

**Figure 9:**
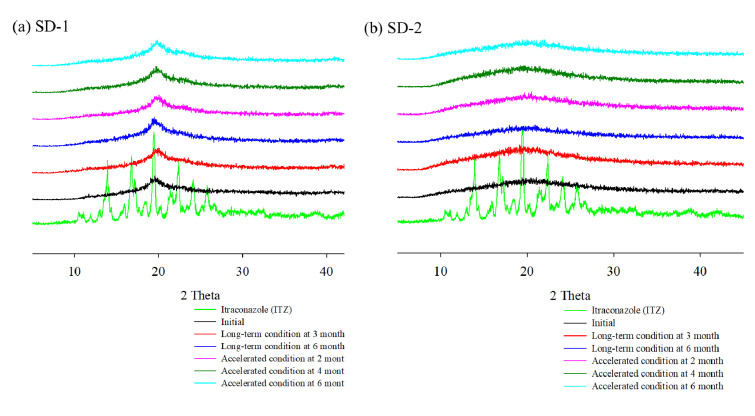
X-ray diffraction of ITZ HME solid dispersions during stability test.

Table 1 and Figure 8 show that there were no significant changes in the drug content and dissolution rates for the SD-1 and SD-2 formulations under long-term (25 ± 2 °C, 60% ± 5% RH) and accelerated (40 ± 2 °C, 75% ± 5% RH) storage conditions. There were no significant differences in the drug content or release behavior of ITZ between the initial stage of the SD and its condition after 6 months. In addition, PXRD analysis was performed to evaluate the changes in the crystal form during the stability test (Figure 9). Diffraction peaks of the crystalline ITZ in SD-1 and SD-2 did not appear under both storage conditions, confirming the stability of the SDs that were prepared using HME.

### 2.4. In Vivo Studies

**Figure 10:**
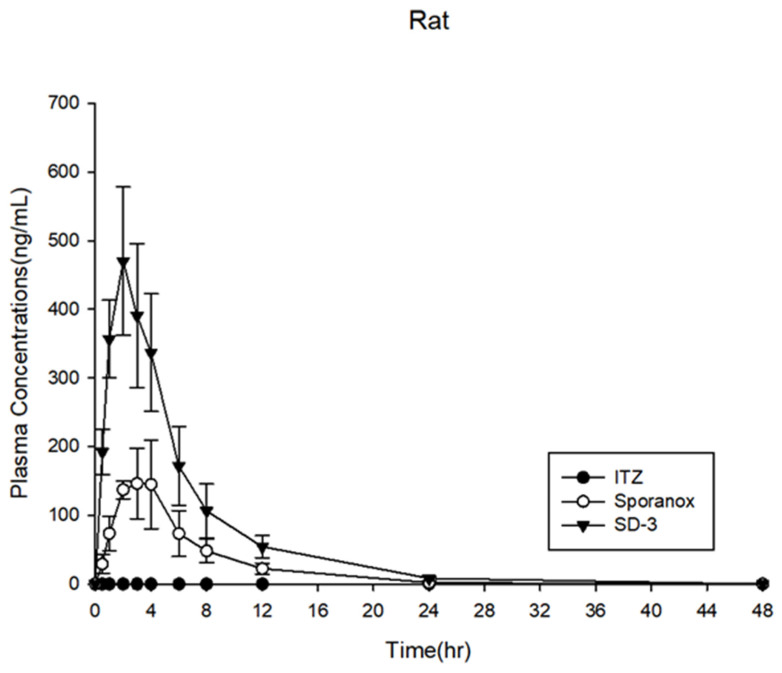
Plasma concentration profiles in rats (*n* = 6).

The PK profiles in the rats are shown in Figure 10, while the PK parameters are shown in Table 2. Crystalline ITZ was not detected in the plasma because of its low solubility and bioavailability. Sporanox^®^ reached the maximum blood concentration at 2.8 h (T_max_), with a C_max_ of 167.8 ± 53.4 ng·mL^−1^ and AUC_0–48h_ of 1073.9 ± 314.7 ng·h·mL^−1^. The T_max_, C_max_, and AUC_0–48h_ of the ITZ in SD-3 were 2.0 h, 1073.9 ± 314.7 ng·mL^−1^, and 2969.7 ± 720.6 ng·h·mL^−1^, respectively. These results showed that the ITZ AUC_0–48h_ of the SD-3 capsules was three-fold higher than that of Sporanox^®^. However, in the SD-3 capsule-administered group, it was expected that SD-1 would be released in the stomach under acidic conditions to create supersaturation, and SD-2 would be released at a neutral pH. Although a bimodal PK profile was expected, the PK profile of the SD-3 capsule showed only a single peak. Therefore, in vivo assessments were also performed in beagle dogs to investigate the release mechanism of the ITZ SDs.

**Figure 11:**
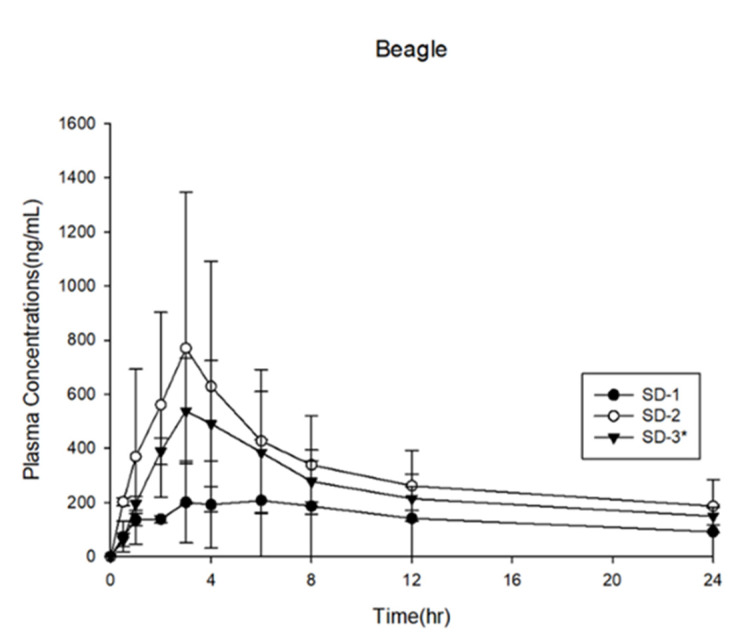
Plasma concentration profiles in beagle dogs (*n* = 3).

The SD-1, SD-2, and SD-3 capsules were prepared and administered to beagle dogs. The results of the in vivo analysis performed in beagle dogs are shown in Figure 11 and Table 3. The AUC_0–24h_ of SD-1 was 3.37 ± 3.28 μg·h·mL^−1^, and that of SD-2 was 7.50 ± 4.50 μg·h·mL^−1^, indicating that the AUC_0–24h_ of SD-2 was 2.2 times higher than that of SD-1 in the beagle dog model. The dissolution rate of SD-1 was significantly higher than that of SD-2 at pH 1.2. The ITZ in the SD-1 formulation was dispersed only in the PVA-based Parteck^®^ MXP. The solubility of ITZ in a binary SD system is easily affected by the external environment, such as the pH of the simulated biological fluid. As a result, the mean plasma concentration of ITZ in SD-1 was lower than that in SD-2.

In contrast, the HPMCP-HP55 and Soluplus^®^ in the SD-2 formulation could have sterically inhibited the recrystallization of ITZ because HPMCP-HP55 could dissolve at pH 5 or even higher at pH 6. The precipitation of the BCS class II drug, ITZ, which exhibited dissolution-rate adsorption, could be synergistically inhibited by polymer-based surfactants [41]. The in vivo adsorption area of ITZ was narrow. Considering the ITZ dissolution rate and plasma concentration of SD-2, which had low solubility in gastric fluids, these results indicate that the plasma concentration of ITZ was highly affected by the solubility of ITZ in the dissolution medium. By adjusting the proportion of the ITZ SD pellets with different compositions, it was possible to achieve a target dissolution rate in a narrow absorption area.

The SD-3 pellets consisted of a 50% mixture of SD-1 and SD-2. The PK profile of SD-3 exhibited a plasma concentration of ITZ that was between those of SD-1 and SD-2. The in vivo experiment was performed with the animals in a fasted state, and the pH of the gastric fluid in the fasting beagle dog was approximately 2.0, which is higher than the actual pH that was used in the in vitro *dissolution* experiment [42]. Furthermore, Yoo et al. (2000) reported that the dissolution rate of Sporanox^®^ was 86.5 ± 1.8% at pH 1.2 and 18.9 ± 3.97% at pH 2.0 in 60 min. A slight increase in pH from 1.2 to 2.0 substantially reduced the dissolution rate of ITZ four-fold [41]. According to a US patent (US patent no. 9,492,446 B2, 2016), the solubility of the patented ITZ SD that was produced using a spray–dry technique with PVA and ITZ (in a ratio of 1:1) decreased by 5.6 times when the pH of the medium was changed from 1.6 to 2.4. Therefore, it is highly recommended that ITZ is administered immediately after a meal to improve its bioavailability [43,44].

SD-2 was not released at a low pH but was released and absorbed in the duodenum. The bioavailability of SD-2 was three times higher than that of SD-1. Actually, ITZ was absorbed in a supersaturated state in the absorption area [45,46,47]. Based on these in vivo results, we confirmed that the bioavailability of ITZ increased when supersaturation occurred in the small intestine. In addition, the SD-2 pellet showed enhanced bioavailability, with enhanced solubility and greater inhibition of ITZ precipitation in the absorption area.

## 3. Materials and Methods

### 3.1. Materials

ITZ (USP grade) was purchased from SK Chemical (Seoul, Korea). PVA (Parteck^®^ MXP, average molecular weight: ~32,000) was supplied by Merck KgaA (Darmstadt, Germany). Triethyl citrate (≥99%, molecular weight: 276.28) was purchased from Sigma-Aldrich (Seoul, Korea). Polyvinyl caprolactam-polyvinyl acetate-polyethylene glycol graft copolymer (PCL-PVAc-PEG, Soluplus^®^, average molecular weight: ~118,000) was supplied by BASF (Ludwigshafen, Germany). HPMCP-HP55 (hypromellose phthalate, average molecular weight: ~45,580) was obtained from Shin-Etsu Chemical (Tokyo, Japan). All chemicals and solvents used were of analytical grade.

### 3.2. Preparation of SD Pellets Using HME Technology

Mixtures of ITZ–Parteck^®^ MXP and ITZ–Soluplus^®^ HPMCP-HP55 were prepared using the composition that is summarized in Table 4. The HME SDs were produced using the Pharma 11 Twin-screw Extruder system (Thermo Fisher Scientific, Waltham, MA, USA). In both formulations, 10% triethyl citrate was used as a plasticizer. SD-1 and SD-2 extrudates were pelletized using a VariCut Pelletizer (Thermo Fisher Scientific) at L2–3 speed, resulting in a pellet thickness of 0.5 mm. The weight of the PVA (Parteck^®^ MXP) was conventionally required 70(*w*/*w*)% as per the total weight when the degree of PVA hydrolyzation significantly impacts the inhibition of poorly water-soluble drug precipitation. As considering the drug-polymer miscibility, 80(*w*/*w*)% of Parteck^®^ MXP is enough to act as a strong precipitation inhibitor for the ITZ. Even the 20 *w*/*w*% of HPMCP can provide the modulated release behaviors in different pH environments. The ratios of Soluplus to HPMCP-HP55 were determined by achieving the suitable miscibility for maintaining the assay of the ITZ in the HME process.

### 3.3. Determination of Drug Content in SDs

The drug content in the SDs was evaluated as follows. A standard solution that contained 10 mg of ITZ in 100 mL of mobile phase (acetonitrile: phosphoric acid buffer solution (pH 2.0) = 65:35 *w*/*w*) was mixed using an Ultrasonic Ben 5510 DTH sonicator (Branson, Danbury, CT, USA) for 15 min. If the miscibility between the drug and the polymer is low, then the content uniformity of the drug in the HME formulation would drop significantly during the process. Additionally, the distribution and homogeneity of the drug in the HME formulation could be evaluated using spectroscopic imaging techniques [48,49]. In fact, the compatibility between the drug and the polymer can be the critical parameter for maintaining the assay of the drug. The 0.1 mg/mL of ITZ in the mobile phase was a stock solution for quantitatively analyzing the assay of ITZ in the SD formulation. The test samples (SD-1 and SD-2) were prepared using the same concentration of ITZ as per the standard solution. The samples were passed through a 0.45 μm filter paper. After removing gas from the solution using a sonicator, an Agilent 1200 series HPLC (Agilent Technologies, Santa Clara, CA, USA) system was used to analyze the samples under the following settings: UV detection wavelength, 261 nm; flow rate, 1.0 mL·min^−1^; column temperature, ambient; injection volume, 20 μL; and column, C_18_ (5 μm, 150 mm × 4.6 mm).

### 3.4. Drug Release

Dissolution studies of ITZ PMs and ITZ SDs (with an ITZ amount equivalent to 100 mg) were performed using HME with the paddle apparatus (USP Method II). The ratio of PMs that contained ITZ was equal to the compositions of the SDs. ITZ PMs were prepared by homogeneously mixing the excipient and raw ITZ using a 60-mesh sieve. The dissolution test was conducted with 750 mL of 0.1 N HCl (pH 1.2) for 2 h. The pH of the solution was then adjusted to 6.8 by adding 250 mL of pre-heated 0.2 M trisodium phosphate (Na_3_PO_4_) solution, following which the dissolution test was continued for another 2 h [27]. The dissolution test was performed using a PTWS–121C (Pharma Test, Hainburg, Germany) with the paddle method indicated in USP Apparatus II. During the dissolution test, the temperature was set to 37 ± 0.5 °C, and the paddle speed was set to 75 rpm. Five milliliters of the samples were collected by filtering through a 0.45-µm microfilter at the time points of 5, 15, 30, 45, 60, 90, and 120 min for each pH condition and subjected to HPLC analysis, as described above.

### 3.5. Recrystallization Behavior of ITZ in pH 6.8 Medium

After the dissolution test, the dissolution medium that contained SD-1, SD-2, and Sporanox^®^ was passed through a 200-mesh sieve (75 µm) and transferred to a beaker. It was dried at 60 °C until the medium evaporated. The XRD patterns of ITZ in SDs and Sporanox^®^ were evaluated to understand the recrystallization behavior of ITZ upon changing the pH of the dissolution medium.

### 3.6. Physicochemical Properties of SDs

FE-SEM, DSC, XRD, and FT-IR analyses were performed to determine the morphological and crystalline characteristics of the ITZ SDs. A JSM-6510 (JEOL, Tokyo, Japan) was used for the SEM analysis, at 20 kV and a magnification of 55×–1000×, after the samples were coated with a thin layer of gold for 10 min. A DSC Q2000 (TA Instruments, New Castle, DE, USA) was used for the DSC. Approximately 5 mg of the sample was weighed in a standard open aluminum pan, and the temperature was increased at a rate of 10 °C·min^−1^ from 0 °C to 200 °C, with nitrogen as the purge gas, and the temperature and heat flow calibrated with indium. For the XRD analysis, we used a D8-ADVANCE (Bruker, Billerica, MA, USA) with Cu-Kα radiation (1.5406 Å), which was gently placed in an aluminum holder at 40 kV and 40 mA with a 2-theta value of 5–40° and a scanning speed of 1.2°·min^−1^. The interaction between the ITZ and the polymers was identified using FT-IR analysis. FT-IR analysis was performed using a SINCO IR 200 (Thermo Fisher Scientific), and measurements were taken over a wavelength range of 400–4000 cm^−1^.

### 3.7. Stability Test

The SD-1 and SD-2 pellets were filled into capsules and then placed in a well-closed container under specific conditions for long-term (25 ± 2 °C, 60 ± 5% RH) and accelerated (40 ± 2 °C, 75 ± 5% RH) stability tests. The long-term stability test was conducted at 0, 3, and 6 months and the accelerated test was conducted at 0, 2, 4, and 6 months. The drug content, release rate, and XRD patterns of the samples were evaluated at each time point.

### 3.8. In Vivo Studies

In vivo studies were performed using rats and beagle dogs to confirm the enhancement of bioavailability. The major absorption areas of ITZ can be identified in the different gastrointestinal systems of each biological species. The gastrointestinal system was differentiated according to its length and diameter. Through allometric scaling, these PK data could be correlated with those of human and animal species. Furthermore, the in vivo performance of ITZ, depending on the animal species, could be predicted through dose estimation.

An in vivo rat model assessment was used to investigate the absorption behavior of ITZ. Three test groups were administered to rats: SD-3 (50% SD-1 and 50% SD-2 pellets), ITZ crystalline powder, and Sporanox^®^. ITZ crystalline powder and Sporanox^®^ (ITZ 100 mg, Janssen) were used as reference formulations. The mean weight of the 18 rats (male Sprague–Dawley rats) was 276.46 ± 8.51 g. The rats were divided into three groups of six rats each. SD-3 pellets, Sporanox^®^, and ITZ crystalline powder were milled with a mortar and pestle and suspended in purified water that contained citric acid and xanthan gum. Each ITZ formulation was administered to the rats after a 16 h fast. The feeding resumed 4 h after the administration of ITZ. The rats in all of the groups were administered 20 mg/kg of ITZ. Blood samples (300 μL each) were collected from the jugular vein at 0, 0.5, 1, 2, 3, 4, 6, 8, 12, 24, and 48 h after drug administration and stored in 15 µL heparin-treated 1.5 mL microtubes. The blood samples were centrifuged at 1200 rpm/4 °C for 3 min. After centrifugation, more than 100 μL of the supernatant (plasma) was transferred to a microtube. The plasma samples were then stored at below −70 °C in a deep freezer before quantitative analysis. These animal experiments were performed following the protocol reviewed and approved by the Animal Experimental Ethics Committee of the Korea Preclinical Center (KPC-E2019035).

In vivo assessment to evaluate the bioavailability of the SD-1, SD-2, and SD-3 capsules was performed using a beagle dog model. The tested groups of ITZ formulations were administered to male beagle dogs (*Canis familiaris*) (weighing 10.6 ± 0.6 kg, *n* = 3) after 16 h of fasting. The feeding was resumed 4 h after drug administration. The drug administration dose was 100 mg/head, and approximately 3 mL of blood was collected at the time points of 0, 0.5, 1, 2, 3, 4, 6, 8, 12, and 24 h after drug administration. The collected blood samples were stored in 10 µL heparin-treated 1.5-mL microtubes. The supernatant (plasma) obtained after centrifuging the blood samples at 4000 rpm/4 °C for 10 min was divided into two sets of 500 μL each in 1.5-mL microtubes and stored below −80 °C in a deep freezer before being used for quantitative analysis. This animal experiment was performed following the protocol reviewed and approved by the Animal Experimental Ethics Committee of the Korea Preclinical Center (KPC-M2019123).

Non-compartmental analysis was conducted using a WinNonlin (Pharsight Corporation, Mountain View, CA, USA). T_max_, C_max_, *t*½, and lambda z were calculated using the empirical data. The area under the plasma concentration-time curve (AUC_t_) was calculated using the linear trapezoidal method. Statistical analysis to calculate significant differences (α = 0.05) was conducted using a two-tailed Student’s *t*-test.

## 4. Conclusions

An SD pellet containing a poorly soluble drug, ITZ, was successfully prepared using HME technology and a pelletizer. SD-1 was designed to increase the dissolution rate of ITZ in the gastric environment, while SD-2 was designed to increase the same in the upper small intestine. The release rate of SD-2 gradually increased as the pH of the dissolution medium increased from 1.2 to 6.8. However, the bioavailability of ITZ in SD-2 was higher than that in SD-1. SD-1 presented lower bioavailability (by approximately three-fold) than SD-2 in the in vivo studies carried out in beagle dogs because of the pH and the narrow absorption area of the gastrointestinal system. The dissolution rates of the SD pellets increased at the desired pH. Ultimately, the ITZ SD-3 pellets (SD-1 50%, SD-2 50%) also increased at the desired pH. These results indicated that the absorption area where the drug was immediately released to absorb was strongly associated with the bioavailability of ITZ. This study has aimed to improve ITZ absorption by combining HME formulations with conventional processes and compositions. Consequently, the bioavailability of poorly water-soluble drugs could be modulated by maintaining the supersaturated state of the model drug. As the pH solubility of a drug should be considered according to the absorption window of the drug, this formulation strategy can be a promising option for the poorly water-soluble drug.

## Figures and Tables

**Table 1:** Content of itraconazole in the solid dispersions during the stability tests (unit: percentage).

Time and Stability Testing Conditions	SD-1	SD-2
Initial	99.20 ± 2.51	98.43 ± 3.11
Long-term 3 months	97.30 ± 1.71	95.41 ± 3.66
Long-term 6 months	98.83 ± 0.93	96.71 ± 2.21
Accelerated 2 months	97.15 ± 2.00	98.55 ± 1.15
Accelerated 4 months	97.15 ± 2.71	98.31 ± 1.05
Accelerated 6 months	97.97 ± 1.39	97.90 ± 1.91

**Table 2:** Pharmacokinetic parameters of itraconazole in rats (*n* = 6).

Parameter	ITZ	Sporanox^®^	SD-3
C_max_ (ng·mL^−1^)	None	167.8 ± 53.4	469.8 ± 108.0
T_max_ (h)	None	2.8 ± 1.0	2.0 ± 0.0
AUC_0–48h_ (ng·h·mL^−1^)	None	1073.9 ± 314.7	2969.7 ± 720.6

**Table 3:** Pharmacokinetic parameters of itraconazole in beagle dogs.

Parameter	SD-1	SD-2	SD-3
C_max_ (ng·mL^−1^)	0.26 ± 0.18	0.77 ± 0.58	0.55 ± 0.18
T_max_ (h)	3.0 ± 2.8	3.0 ± 0.0	2.5 ± 0.7
AUC_0–24h_ (ng·h·mL^−1^)	3.37 ± 3.28	7.50 ± 4.50	6.05 ± 1.71

**Table 4:** Composition of solid dispersions prepared using HME technology.

Formulation and Condition	SD-1	SD-2
Itraconazole (ITZ)	20%	20%
Parteck^®^ MXP	80%	0%
Soluplus^®^	0%	60%
HPMCP-HP55	0%	20%
Processing parameter	SD-1	SD-2
Screw speed (RPM)	50	30
Processing temperature (°C)	200	170

## Data Availability

Data is contained within the article.
